# Lipoic acid decreases Mcl-1, Bcl-x_L_ and up regulates Bim on ovarian carcinoma cells leading to cell death

**DOI:** 10.1186/s13048-015-0165-z

**Published:** 2015-06-12

**Authors:** Perrine Kafara, Philippe Icard, Marilyne Guillamin, Laurent Schwartz, Hubert Lincet

**Affiliations:** INSERM, U 1199, Unité BioTICLA, Caen, F-14032 France; Normandie Université France, Caen, France; Centre de Lutte Contre le Cancer François Baclesse, Avenue du Général Harris, Caen, BP5026 14076 Cedex 05, France; UMR 7161, Laboratoire d’Informatique LIX, Ecole Polytechnique, 91128 Palaiseau Cedex, France; UMR INSERM U1052, CNRS 5286, Centre de Recherche en Cancérologie de Lyon, Lyon, France; ISPB, Faculté de Pharmacie de Lyon, 8 avenue Rockefeller, F69373 Lyon, Cedex 08 France; Université de Lyon 1, Lyon, France

**Keywords:** Lipoic acid, Mcl-1, Bcl-x_L_, Bim, ovarian cancer, ROS

## Abstract

**Background:**

Ovarian carcinoma is the leading cause of death from gynecological cancer because there is risk of chemoresistance. As previously demonstrated in our laboratory, Alpha-lipoic acid (LA), a co-factor for metabolic enzymes, suppresses the tumor growth. In this study, we have researched the mechanisms that are responsible for the activity of LA.

**Methods:**

We have studied the mechanisms of LA in two ovarian cancer cell lines, a cisplatin sensitive one, IGROV1 and its resistant counterpart, IGROV1-R10. These cells have been exposed to lipoic acid at various concentrations. Cell proliferation, cell cycle repartition and nuclear staining with DAPI were recorded. Western blot analyses were performed to detect various proteins implied in apoptotic cell death pathways. To investigate the formation of ROS, the oxidation of CM-DCFH_2_-DA were also determined.

**Findings:**

LA suppressed growth proliferation and induced apoptosis in both ovarian cell lines. Moreover, LA provoked a down regulation of two anti-apoptotic proteins, Mcl-1 and Bcl-x_L_ protein and a strong induction of the BH3-only protein Bim. Furthermore, LA induced ROS generation which could be involved in the CHOP induction which is known to activate the Bim translation.

**Conclusions:**

Our results reveal novel actions of LA which could explain the anti-tumoral effects of the LA. Therefore, LA seems to be a promising compound for ovarian cancer treatment.

## Findings

### Background

Alpha-Lipoic acid (LA) is a naturally antioxidant lipophilic compound synthesized in small amounts by plants and animals, including humans. It is an essential co-factor for mitochondrial enzymes (e.g. pyruvate dehydrogenase (PDH), succinate dehydrogenase (SDH)) [1, 2] involved in tricarboxylic acid cycle (TCA). Furthermore, LA has antioxidant and redox-regulating properties [3]. The reduced form of LA known as dihydrolipoic acid is the predominant form that interacts with reactive oxygen species (ROS), although the oxidized forms can also inactive free radicals [4]. Beneficial effects of LA in several non tumoral pathologies have been described [5]. In cancer studies, LA suppressed the proliferation of cells such as bladder, breast, colon, hepatoma, and lung [6–11] without effect on normal cells [12] such as liver, ovarian, neurons and hepatocytes [13–15]. These effects could induce the apoptosis that is impaired in cancer.

Apoptosis is under the control of Bcl-2 family members, promoting or inhibiting this process [16]. Death signals activate and/or induce pro-apoptotic members such as Bim which promotes activation of pro-apoptotic effectors (Bax and Bak) and releasing apoptogenic factors from mitochondria [17]. Bim, is one of the most potent BCL-2 homology (BH) 3-only molecules, in terms of cell killing [18], and has also clearly overlapping functions in p53-dependent and p53-independent apoptosis. Anti-apoptotic members, such as Bcl-2, Bcl-x_L_ and Mcl-1, overexpressed in numerous human malignancies, contribute to cell survival and drug resistance [19, 20]. Bcl-x_L_ overexpression has been found associated with chemoresistance and its decrease involved in apoptosis [21, 22]. Moreover, *MCL-1* gene, frequently amplified in human cancers [20], is associated with chemoresistance and relapse [19, 20, 23]. The reduction of Mcl-1 expression leads to apoptosis in numerous cancer cells [22–26]. This reduction is notably induced by glucose privation [27].

Many cancer cells preferentially enhance aerobic glycolysis and transform a significant part of glucose in lactate, even in the presence of oxygen, a common feature of tumor growth described as the Warburg effect [28]. This metabolism furnishes a significant share of ATP and essential intermediates required for tumor proliferation [29]. Its inhibition arrests cancer cell growth [26, 30–32]. The Warburg effect should be in relation with inactivation of PDH and/or over-activation of LDH [29]. The PDH inactivation disconnects TCA from glycolysis, and in place of pyruvate, glutaminolysis replenishes TCA cycle. LA may reactivate PDH and could be a promising molecule to counteract tumor metabolism [2].

In this study, we examined effect of LA on cellular growth of two human ovarian carcinomas and the molecular mechanisms involved. We found LA demonstrated anti-proliferative effect, induced cell cycle arrest and apoptosis. In our model, the anti-tumoral effects of LA might involve, at least partially, from its property to decrease Mcl-1 and Bcl-x_L_ and to up regulate the BH3 only protein Bim through CHOP induction.

### Materials & methods

#### Cell lines and culture conditions

The IGROV1 cell line was kindly provided by Dr. J. Bénard (Institut Gustave Roussy, Villejuif, France). The variant highly chemoresistant cell line, IGROV1-R10, was established as previously described by Poulain *et al.* [33]. Cells were grown in RPMI-1640 medium + Glutamax™ (Gibco Life Technologies, Cergy-Pontoise, France) supplemented with 10 % fetal calf serum, 33 mM sodium bicarbonate (Gibco Life Technologies, Cergy Pontoise, France). Cells were maintained at 37 °C in a 5 % CO_2_ humidified atmosphere and split twice a week by trypsinization.

#### Lipoic acid

Lipoic acid (LA) was purchased from Meda Pharma (Bad Homburg v.d.h, Germany). This compound is preconditioned in a bulb for a volume of 24 ml. This solution contains 600 mg alpha-lipoic acid. The other ingredients are Trometamol (known by its synonym Tris) and water for the injectable. Data were obtained from the supplier. 5.10^5^ cells were seeded in 25 cm^2^ flask day before treatment. When cells have reached their exponentially growing phase, they were treated 24 h later continuous manner. The solution is put directly into the flasks at the concentration studied (0.1; 0.5 and 1 mM).

#### siRNA synthesis and Transfection

All siRNAs used in these studies were chemically synthesised by Eurogentec (Liege, Belgium) and were received as annealed oligonucleotides. The sequence of the double-stranded RNA used to inhibit Bim expression (denoted siBim) is anti-sense 5′-uaacagucguaagauaacctt-3′. Control siRNA (noted siCTRL) was purchased from Eurogentec (Eurogentec Negative Control SiRNA). According to the manufacturer’s instruction, exponentially growing cells were seeded the day before to reach around 50 % confluence at the time of transfection. The transfection has been described by Lepleux *et al.* [31].

#### Proliferation analysis

Cell number and viability were estimated at various times after the beginning of treatment by a semi-automated image-based cell analyzer (Cedex XS Analyser, Roche Applied Science, Meylan, France) using the trypan blue exclusion method*.*

### Analysis of cellular DNA content by flow cytometry

Cells were prepared for flow cytometry as detailed [22, 31]. Briefly, adherent and detached cells were pooled, washed in PBS and fixed in 70 % ethanol, centrifuged then incubated for 30 min at 37 °C in PBS. Pellets were collected and resuspended for staining with Propidium Iodide (PI) using the DNA Prep Reagent Kit (Beckman-Coulter, Villepinte, France). Samples were then analysed using Gallios flow cytometer (Beckman Coulter) equipped with a blue diode at 22 mW. The fluorescence of Propidium Iodide was collected in the FL3 channel with a 620 nm bandpass filter. The doublets were excluded from analysis using an area versus peak DNA content histogram. The singulets were analysed in a single-parameter histogram.

Gallios software was used for data acquisition. Kaluza Software (Beckman Coulter) was run for data analysis.

#### Nuclear morphology study with 4′,6-diamidino-2-phenylindole (DAPI)

After treatment, both detached and adherent cells were pooled after trypsinization, collected on polylysine-coated glass slide by cytocentrifugation, and fixed in ethanol/chloroform/acetic acid solution (6:3:1). The preparations were treated as described by Lepleux *et al.* [31].

#### Western immunoblotting

Adherent cells were rinsed with deionized water and lysed with lysis buffer (pH 8.8 30 mmol.L^−1^ Tris buffer containing 6 mol.L^−1^ urea, 2 mol.L^−1^ thiourea, 2 % CHAPS, 1X protease inhibitor Mix. Western blot were carried out as described [22]. The membrane was either incubated overnight at 4 °C in T-TBS-milk 5 % with the following primary antibodies: anti-Mcl-1 (Santa Cruz Biotechnology), anti-Noxa (Calbiochem), and anti-actin (Millipore). Cleaved and total Caspase 3, PARP, Bcl-x_L_, Bim and CHOP were purchased from Cell Signaling Technology (Ozyme, Saint Quentin en Yvelines, France). Membranes were washed with T-TBS and incubated with anti-rabbit or anti-mouse secondary antibodies. Revelation was carried out using ECL Prime detection reagent (GE Healthcare, Orsay, France).

### RNA extraction and real-time quantitative reverse transcription-PCR (qRT-PCR)

Total RNAs were extracted using the TRIzol® (Invitrogen, LifeTechnologies, Cergy Pontoise, France). The qRT-PCR was carried out as described [34]. Primer and probe sequences for real-time detection of Bim mRNA (assay ID#Hs00708019_s1), Mcl-1 mRNA (assay ID#HS 001 720 36_m1) and endogenous control gene GAPDH mRNA [34] were purchased form Applied Biosystems. Bim and Mcl-1 transcripts were quantified relative to GAPDH and normalized to control untreated cells by the comparative 2^ΔΔct^ method.

### Measurement of ROS with CM-DCFH_2_-DA probe

The probe CM-DCFH2-DA was used for assess the production of ROS according to manufacturer’s instructions. The CM-DCFH2-DA passes through the cell membrane and once inside the cell is converted to the non-fluorescent derivate dichlorofluorescein, which in turn remains inside the cell and reacts with intracellular ROS to produce the DCF. To study the time-dependent effect of α-LA on ROS production, 5.10^5^ cells were exposed to 0.5 or 1 mM of α-LA for 3 h, 6 h or 24 h, and were incubated with 5 μM CM-H_2_DCFDA (C6827, Molecular probes) for 30 min at 37 °C in dark. The ROS scavenger NAC (3 mM) (Sigma-Aldrich, France) was added 1 h before LA treatment (1 mM). After, adherent cells were washed out the excess probe with PBS and then trypsinizated. The fluorescence of DCF was measured in the FL1 channel with a 525 nm bandpass filter on Gallios flow cytometer (Beckman Coulter). Gallios software was used for data acquisition. Kaluza Software (Beckman Coulter) was run for data analysis. The results were treated and presented as means ± standard errors of the means (SEM) of three independent experiments using GraphPad Prism5 software.

### Results

#### Effects of lipoic acid on cell growth and cell cycle

**Fig. 1:**
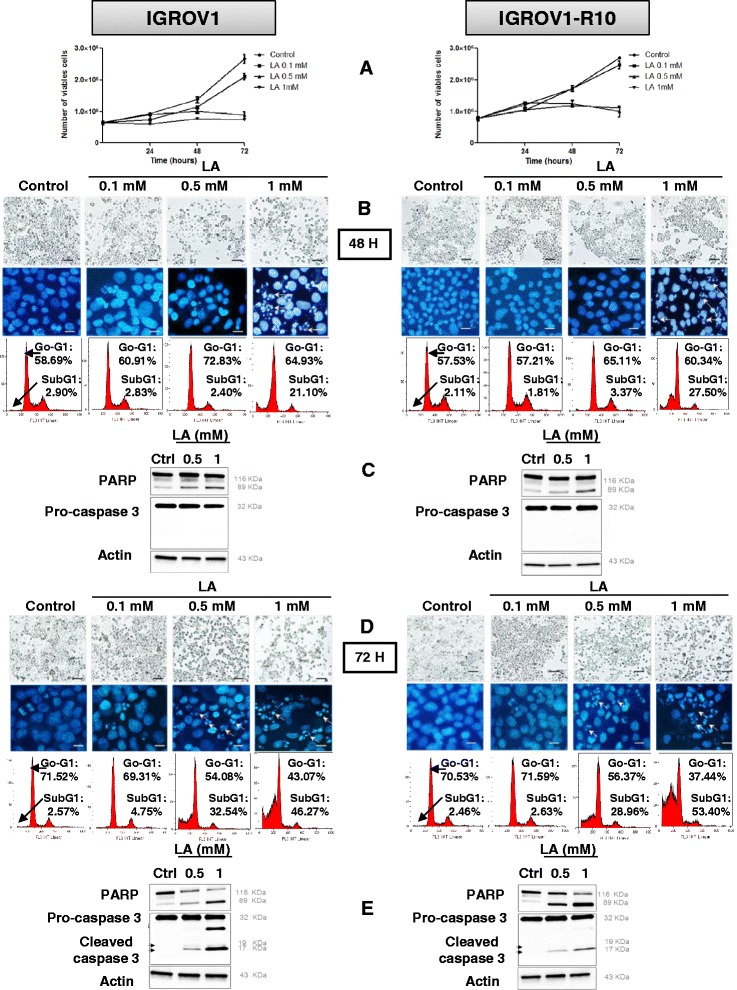
Lipoic acid induces apoptosis in IGROV1 and IGROV1-R10 ovarian carcinoma cell lines. IGROV1 (left panel) and IGROV1-R10 (right panel) were treated to a continuous exposure to 0.1; 0.5 and 1 mM of lipoic acid (LA) and effects of this treatment were analyzed after 48 h and 72 h. **a**: Cell Viability was expressed as number of viable cells determined by the trypan blue exclusion method. Graphics were realized and are presented as means ± standard errors of the means (SEM) of three independent experiments using GraphPad Prism5 software. **b** and **d**: Morphological features of the cells observed by photon microscopy (upper line of each panel) and nuclear features of the cells after DAPI staining (middle line of each panel) were then studied, Bars: 20 μm. DNA content histograms obtained by flow cytometry (lower line of each panel) after a 48 h treatment (**b**) or a 72 h treatment (**d**). For each condition, the percentage in sub-G1 and G0-G1 phases is indicated. **c** and **e**: Protein expression levels of PARP (native and cleaved forms), caspases-3 (pro and cleaved forms) were assessed in control or LA-treated (0.5 or 1 mM) cells at 48 h (**c**) and 72 h (**e**) by western blot using a specific anti-PARP and anti-caspase-3 antibody. Expression of actin was measured as a loading control. Western blots shown are from one experiment representative of at least three independent experiments and cell lysates

To explore the therapeutic potential effect of LA for ovarian cancer treatment, we evaluated its effect on cell growth and cell cycle distribution. For studying cellular growth, Cisplatin-sensitive (IGROV1) and cisplatin-resistant (IGROV1-R10) human cell lines, were cultured either in absence or in presence of LA (0.1, 0.5 and 1 mM). In each cell line, cell growth was significantly reduced in a dose-dependent manner, whereas control cells increased 3–4 folds (Fig. 1a). LA (1 mM) strongly inhibited cell growth at each time point tested, and the number of viable cells remaining close to seeding after 72 h (Fig. 1a). At 0.5 mM, this inhibition was also clear, but delayed after 48 h treatment. At this time, we studied cellular morphology, distribution in the different phases of cell cycle and DAPI nuclear staining. At LA 0.1 mM, no effect was detected in both cell line (Fig. 1b). From LA 0.5 mM, IGROV1 and IGROV1-R10 cells were less confluent, as compared to untreated cells or treated with LA 0.1 mM (Fig. 1b, upper line of each panel). IGROV1 and IGROV1-R10 showed an important percentage of cells in sub-G1 phase (21.1 %, and 27.5 % respectively) in response to LA 1 mM (Fig. 1b, lower line of each panel). These results were confirmed by DAPI nuclear staining which showed more features of cell death, e.g. nuclear condensations and fragmentations (as named apoptotic bodies) (Fig. 1b, middle line of each panel) and also by PARP cleavage which has been found but not a caspase-3 cleavage from 0.5 mM in IGROV1 and resistant counterpart IGROV1-R10 cells (Fig. 1c).

After 72 h of treatment (0.5 and 1 mM), in each cell line, many rounded cells were detected, suggesting detached cells; a phenomenon which seemed dose-dependent (Fig. 1d, upper line of each panel). The cell cycle repartition revealed that percentage of cells in sub-G1 phase is drastically increased at 72 h as compared to 48 h in both cell lines and is also increased in a concentration-dependent manner reaching 53.4 % at LA 1 mM (Fig. 1d, lower line of each panel). Moreover, DAPI staining revealed numerous apoptotic bodies after exposure to 0.5 or 1 mM LA (Fig. 1d middle line of each panel). At this time, we can see that a caspase-3 activation which was accompanied by a strong decrease of the full length PARP (116 kDa) (Fig. 1e).

These results indicated that LA inhibited cellular proliferation and induced a sub-G1 peak associated caspase-3 activation. The cell detachment observed in flasks, might be associated with cell death and deregulation of apoptosis-related proteins.

### Effect of lipoic acid on expression of the apoptosis-related proteins

**Fig. 2:**
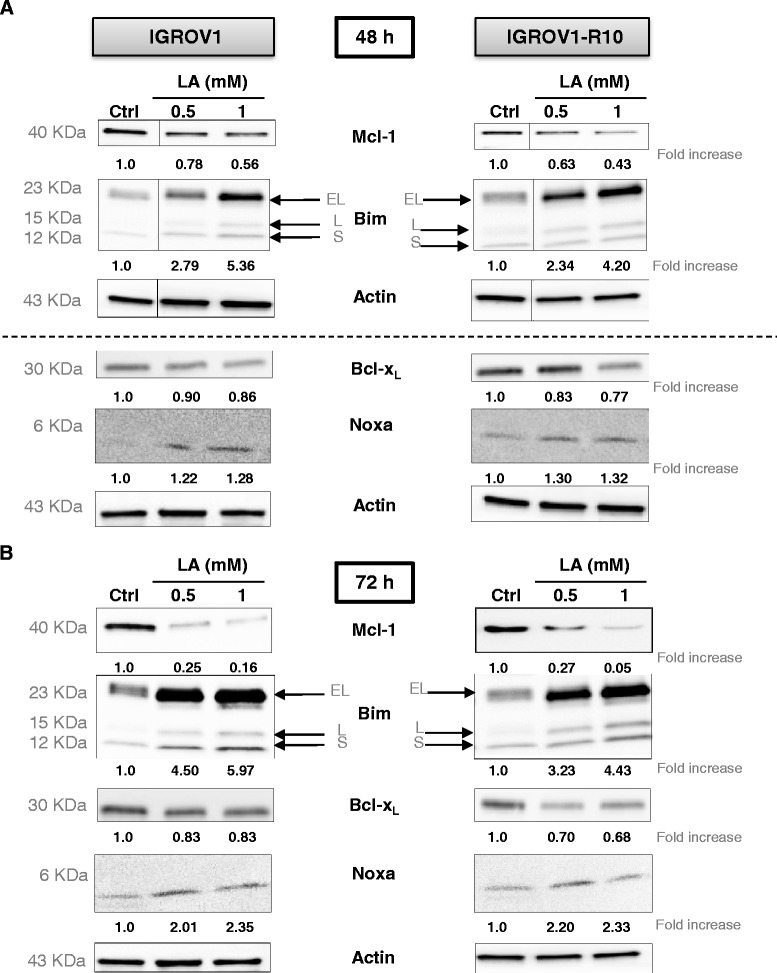
Lipoic acid modulates Bcl-2 proteins family expressions. After 48 h (**a**) or 72 h (**b**) of LA treatment on IGROV1 (left panel) and IGROV1-R10 (right panel) cell lines, whole cell lysates were immunoblotted for the indicated proteins and actin was loaded as control. The relative densitometry values were quantified by Image J® software and are shown on the bottom. Western blots shown are from one experiment representative of at least three independent experiments and cell lysates. Moreover, on Fig. 2a, there are two different strips of actin both blots were carried out independently of one another

We tested whether the effect of LA (0.5 or 1 mM) was associated with a modulation the expression of the apoptosis-related proteins, Bcl-x_L_, Mcl-1 and Bim. At 24 h after exposure to LA, no protein variation was detected (data not shown). Anti-apoptotic Mcl-1 protein level was reduced in a concentration-dependent manner after a 48 h exposure to LA 0.5 - 1 mM in both cell lines (Fig. 2a upper panel) and Mcl-1 expression was almost abolished after 72 h exposure to the LA concentrations (Fig. 2b lower panel), and was concomitant with the induction of apoptosis (PARP cleavage, caspase-3 activation) (Fig. 1e). For the same conditions, a concomitant reduction of Bcl-x_L_ level was found (around 20-30 %) (Fig. 2b lower panel).

Interestingly, Bim was induced by LA in a dose and time-dependent manner with occurrence of BimEL and BimS forms at 72 h (Fig. 2a and b).

**Fig. 3:**
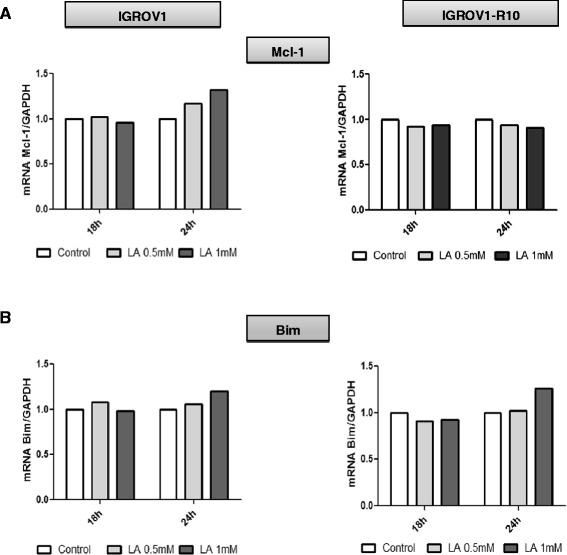
Lipoic acid regulates Mcl-1 and Bim mRNA levels. Mcl-1 (**a**) and Bim (**b**) mRNA levels in IGROV1 (left panel) and IGROV1-R10 (right panel) treated with LA different times was assessed by real-time quantitative reverse transcription PCR. Data are normalized with GAPDH mRNA levels used as an endogenous control. Results are expressed as relative to the levels in control cells set as one. This analysis was performed only 2 times

To determine whether the inhibition of Mcl-1 protein and the increase of Bim were due to a reduction of mRNA, we performed qRT-PCR. Whatever the time and the LA concentration, no decrease in Mcl-1 mRNA was observed (Fig. 3a). On the contrary, LA induced a low up-regulation of Bim mRNA in both cell lines (Fig. 3b).

### siRNA-mediated Bim inhibition decreases the cell death in response of lipoic acid

**Fig. 4:**
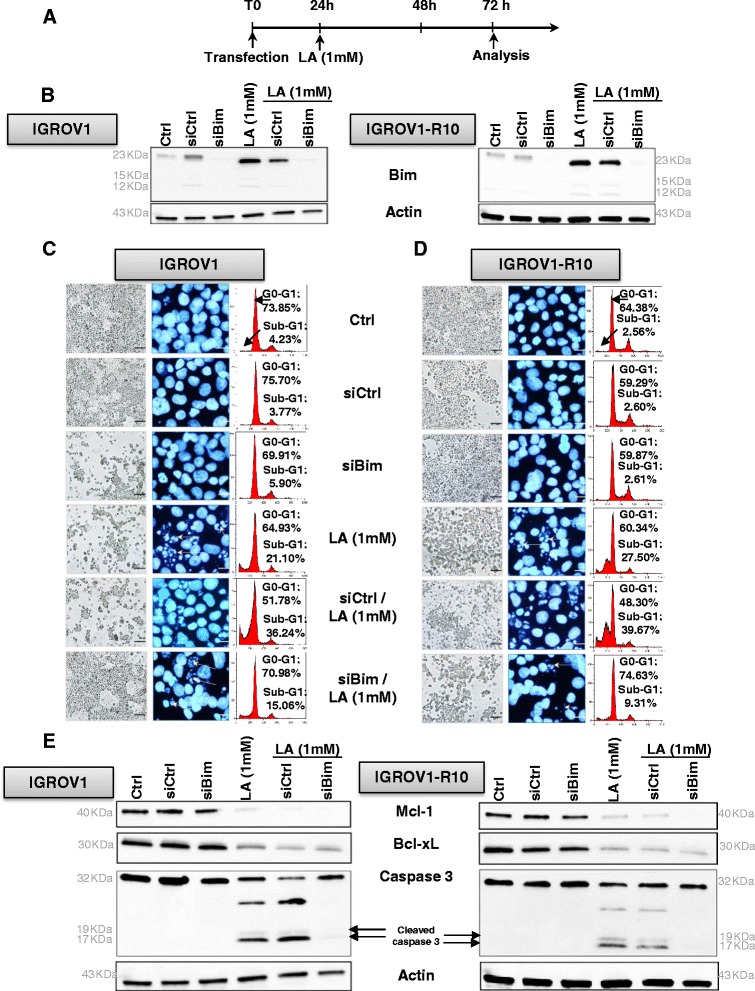
siBim attenuates lipoic acid induced-apoptosis 72 h after transfection. **a**: The cells were treated following protocol of exposure regarding the treatment by lipoic acid (1 mM) administered 24 h after transfection with either 20nM nonspecific siRNA control (siCRTL) or siBim, as described in materials and methods section. **b**: Bim protein expression level was assessed in control or treated-cells at 72 h post-transfection of IGROV1 (left panel) and IGROV1-R10 (right panel) by western blot. Actin protein is used as a loading control. Actin is a same actin that in Fig. (4e). This blot was performed in the same experiment as that of blot in Fig. 4e. Western blots shown come from one experiment representative of at least three independent experiments and cell lysates. **c**-**d**: Morphological features of the cells observed by photon microscopy (left column of each panel) and nuclear features of the cells after DAPI staining (middle column of each panel) were then studied, Bars: 20 μm. DNA content histograms obtained by flow cytometry (right column of each panel) after a 48 h of LA treatment in IGROV1 [c] and IGROV1-R10 (**d**) cell lines were studied. For each condition, the percentage of sub-G1 and G0-G1 phases is indicated. **e**: Bcl-x_L_, Mcl-1, caspases-3 (pro and cleaved forms) protein expression levels were assessed in control or treated-cells at 72 h post-transfection of IGROV1 (left panel) and IGROV1-R10 (right panel) by western blot. Actin protein is used as a loading control. Actin is a same actin that in Fig. (4b). This blot was performed in the same experiment as that of blot in Fig. 4b. Western blots shown are from one experiment representative of at least three independent experiments and cell lysates

To study the importance of Bim in the response of our model to LA, we tested the impact of Bim targeting by siRNA on cell death of IGROV1 and IGROV1-R10, 24 h after transfection exposed to LA 1 mM (Fig. 4a). We first checked the efficacy by showing a complete extinction of this protein in both cell lines after 48 h of siRNA transfection. In contrary, Bim expression was not modified in transfected cells with control siRNA (siCTRL) (Fig. 4b).

siBim combination with LA (1 mM) abrogated the cytotoxic effect of LA. Indeed, 48 h after treatment, a decrease percentage of cells in sub-G1 peak, reaching 15 % for IGROV1 and 9 % for IGROV1-R10 was observed when Bim was silenced, as compared to cells treated with LA alone, (Fig. 4c right column of each panel and Fig. 4d right column of each panel).

The DAPI nuclear staining confirmed these results, showing in both cell lines that exposure with siBim and LA 1 mM, strongly decreased these nuclear characteristics of apoptosis (nuclear condensations and fragmentations), which were strongly observed in response to LA treatment alone (Fig. 4c middle column of each panel and Fig. 4d middle column of each panel).

We investigated the impact of targeting Bim by siRNA on the apoptosis induction in our models 48 h after LA treatment. In response to siBim and LA exposure, we observed an inhibition of caspase-3 cleavage and also a decrease of Mcl-1 and Bcl-x_L_ proteins (Fig. 4e). These results demonstrate that Bim is involved in LA-mediated apoptosis despite a decrease of two anti-apoptotic proteins in IGROV1 and IGROV1-R10 cells.

### Lipoic acid may induced the Endoplasmic Reticulum stress response and accumulation of ROS

**Fig. 5:**
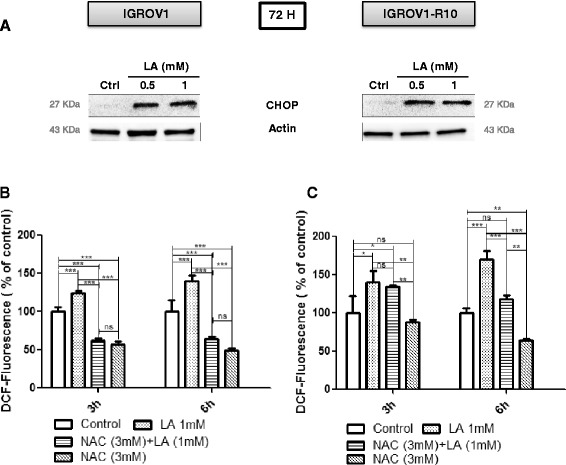
Lipoic acid induces ER stress and increases ROS generation in IGROV1 and IGROV1-R10. **a**: ER stress protein expression was assessed in IGROV1 (left panel) and IGROV1-R10 (right panel) cell lines by immunoblot using an antibody which recognizes CHOP protein. The blot shown is representative of three independent experiments and cell lysates. **b**-**c**: ROS production was measured by flow cytometry using CM-DCFH2-DA probe. Histograms show that 3 or 6 h of LA treatment (1 mM) increased ROS production compared to control and ROS scavenger (NAC). Bars represent the mean of *n* = 3 independent biological replicates ± SEM. Graphics were realized and are presented using GraphPad Prism5 software. ROS levels in treated *vs* no treated cells in IGROV1 or IGROV1-R10 were analyzed by One-way analysis of variance (ANOVA) with post hoc analysis using Newman-Keuls multiple comparison test was used for parametric data. A *p* value of <0.05 was considered statistically significant. * *p* < 0.5; ** *p* < 0.1; *** *p* < 0.01

Because Bim is frequently induced by Endoplasmic Reticulum (ER) stress response via the C/EBP homologous protein (CHOP) expression, we investigated this pathway in response to LA by detecting CHOP. CHOP was strongly expressed at 72 h in IGROV1-R10 from LA 0.5 mM (Fig. 5a right panel) and was correlated with the induction of Bim expression (Fig. 2b right panel).

In order to determine whether LA treatment generates the ROS in our models, we measured by a converting reaction of DCFH_2_-DA to DCF 3 h and 6 h after LA exposure.

At 0.5 mM LA, we measured a slightly increased ROS generation in IGROV1 and IGROV1-R10 cells from 3 h and more important at 6 h in IGROV1-R10 *vs* IGROV1 cells (data not shown). However, the ROS production was more efficient at 1 mM LA whatever the time after treatment (e.g. at 6 h 140 % in IGROV1 and 170 % in IGROV1-R10) (Fig. 5b-c). Interestingly, pretreatment with ROS scavenger NAC (3 mM) decreased α-LA-induced ROS (Fig. 5b-c).

### Discussion

Ovarian cancer is the fifth most frequent cause of cancer death in women, often diagnosed at an advanced stage. Despite radical surgery and a frequent good response to a first-line platinum-based chemotherapy, the 5-year survival rate is about 20 % - 30 % for stage III and IV disease [35]. Thus, discovery of new treatments is fundamental to overcome chemoresistance and improve survival. In this perspective, lipoic acid (LA) molecule which demonstrated a low toxicity in various pathologies (in particular, neurological sequels of chemotherapies) could be essential to be tested clinically, if its efficiency is demonstrated in preclinical studies.

In this study, we demonstrated in two human ovarian cancer cells lines, one highly chemoresistant, that LA suppressed the proliferation and induced the cell death in both cell lines by decreasing of Mcl-1 and Bcl-x_L_ expression, two anti-apoptotic proteins; whereas the induction of CHOP could promote Bim transcription.

In our study, the inhibition of proliferation was time- and concentration-dependent, and was in accordance with other studies in breast, neuroblastoma, colon, and bladder cancer cells [6, 9, 11]. We showed that LA treatment provokes the cell detachment in flasks. This phenomenon could be due to down-regulation of β1-integrin expression [36] and reduces the MMPs-2 and MMP-9 activities [37]. Thus, all data show that LA has the high capacity to inhibit cell growth of numerous cancer cell lines.

First, in cellular cycle and morphology studies, we showed that LA treatment resulted in a slight reduction in the growth through the G1 arrest of the cell cycle, a result in agreement with previous studies [7, 10]. The arrest of the cell cycle was accompanied by a cellular accumulation in the sub-G1 phase, which is a characteristic of cell death. The nuclear morphology revealed fragmentation and condensation which are typical of apoptosis. This apoptosis was also supported by PARP and complete caspase-3 cleavages, in particular at 72 h of treatment in both cell lines. However, a more rapid caspase 3 cleaved was observed in Fig. 4e. It is likely that this difference is due to the transfection process. Indeed to make a transfection, the flasks were seeded with 375 000 cells (results presented in Fig. 4e), less than the 500 000 cells required to seed both cells lines for none transfected experiments (Fig. 1c-e). Thus, it is likely that the difference in seeding accelerates the effect of LA. In that sense, the cleavage of caspase 3, was weaker in Fig. 4e, an attenuation that could indicate a cleavage starting at this time.

It is also noteworthy that LA induced from 48 h, a complete down expression of Mcl-1, an anti-apoptotic protein often up-regulated in cancer cells [25, 38, 39]. LA induced also a strong up-regulation of Bim, a BH3-only protein which is essential for apoptosis of various cell types, including epithelial cells, endothelial cells, neurons, and lymphocytes [18, 40]. We next investigated the impact of siRNA-mediated Bim inhibition on the apoptosis of IGROV1 and IGROV1-R10 cells, in response to LA exposure (1 mM). We observed in the Fig. 4b that siCRTL + LA induced a slowly decrease of Bim expression protein in both cell lines whereas there were an increase of cell death (subG1 peak) in both cell lines. This “contradictory” result is very likely due to the transfection process, which weakens some cells, leading a part of them to cell death. In this condition, Bim silencing diminished apoptotic cell death, as observed by the morphological and nuclear features (Fig. 4b-c left column of each panel). Cell cycle analysis by cytometry revealed that siBim followed by LA (1 mM) reduced the drop of cells in sub-G1 peak, as compared LA treatment alone: 15 % *vs* 21.1 % in IGROV1 and 9.3 % *vs* 27.5 % in IGROV1-R10 cells (Fig. 4b-c right column of each panel).

Likewise, Bim silencing is correlated with an absence of activated-caspase-3 and of PARP cleavage after LA exposure. In consequence, this loss of Bim expression seemed to partially protect our ovarian carcinoma cell lines from death. Similarly, a low expression level of Bim was found significantly correlated with poor survival, notably for patients with melanomas [41] and glioblastomas multiform [42].

The up-regulation of Bim under LA treatment prompted us to investigate other anti-apoptotic proteins such Bcl-x_L_ and Mcl-1, but not Bcl-2, which was not expressed in our cells. Both Bcl-x_L_ and Mcl-1 have been implicated to protect ovarian cancer cells from chemotherapy-induced apoptosis [23, 43], their concomitant decrease appearing essential to trigger the cell death [25].

In our study, we observed that LA treatment decreased Bcl-x_L_ expression (around 20-30 % *vs* control cells) more effectively than in the chemoresistant IGROV1-R10 cell lines. This treatment was more effective on the reduction of Mcl-1 expression (around 20-60 %) which was observed earlier, since 48 h after exposure (Fig. 2a upper panel).

The mechanisms which link LA treatment with the decrease of Bcl-x_L_ expression remain to be further studied. However, in order to explain the decrease of Bcl-x_L_ expression, we can hypothesize that this decrease could be due to the inactivation of Akt which induced a down-regulation of one of its target, NF-κB. Indeed, NF-κB expression has been widely observed in diverse tumor types, in response to hyperactivation of Akt [44], and protects cells against cell death through activation of genes such as Bcl-2 and Bcl-x_L_ [45].

In parallel, we explored the role of ROS production in the up-regulation, knowing that LA is known to induce apoptosis via the production of ROS during mitochondrial respiration from 0.5 mM [15, 46]. Because, this ROS production could be involved in the CHOP induction which is known to activate the Bim translation [47], we studied Bim expression. We found a moderately increase in the level of the *bim* mRNA upon treatment with LA (1 mM) (Fig. 3b), although it was difficult to assert if this increase was significant. Another explanation is that the high expression of CHOP and Bim should be due, at least in part, to the stabilization of these proteins, which are not properly degraded. In that sense, ROS could induce a high ER stress, leading to misfolded proteins. These proteins should be degraded in first by the proteasome and thus, Bim and CHOP would be not degraded as rapidly as they are produced. Furthermore, the ROS production is associated with the induction of proteasome activity leading to the down-regulation of anti-apoptotic proteins such as cellular inhibitor of apoptosis protein 1 and 2 (cIAP-1 and −2) and Mcl-1 [48]. Thus, in our conditions, LA slightly increase the ROS production which also might be down-regulate the Mcl-1 expression. ROS have been shown to be initiators and major contributors of endoplasmic reticulum (ER) stress [49]. Whereas numerous investigations revealed that ER stress could be either a cause, or a result, of increased ROS generation [50], we investigated if the CHOP, a pro-apoptotic transcription factor, was induced by ER stress [51]. We observed that LA treatment highly induced CHOP, which is known to bind an element in the promoter of the gene encoding Bim protein [47]. This induction of CHOP was associated with concomitant Bim and Noxa up-regulation (Fig. 2a-b), two factors crucial for induction of executing apoptosis [50, 52, 53]. Because LA induced Mcl-1 down-regulation and knowing that ER stress was known to induce Bim and Noxa upregulation [53]. Then, we investigated Noxa expression. In response to LA exposure, we observed an induction of Noxa in dose and time-dependent manner. This expression of Noxa is in line with CHOP expression associated with ER stress [53]. Moreover, the PERK protein plays a major role in tethering the ER to mitochondria thereby promoting the rapid transfer of ROS signals [51, 53].

**Fig. 6:**
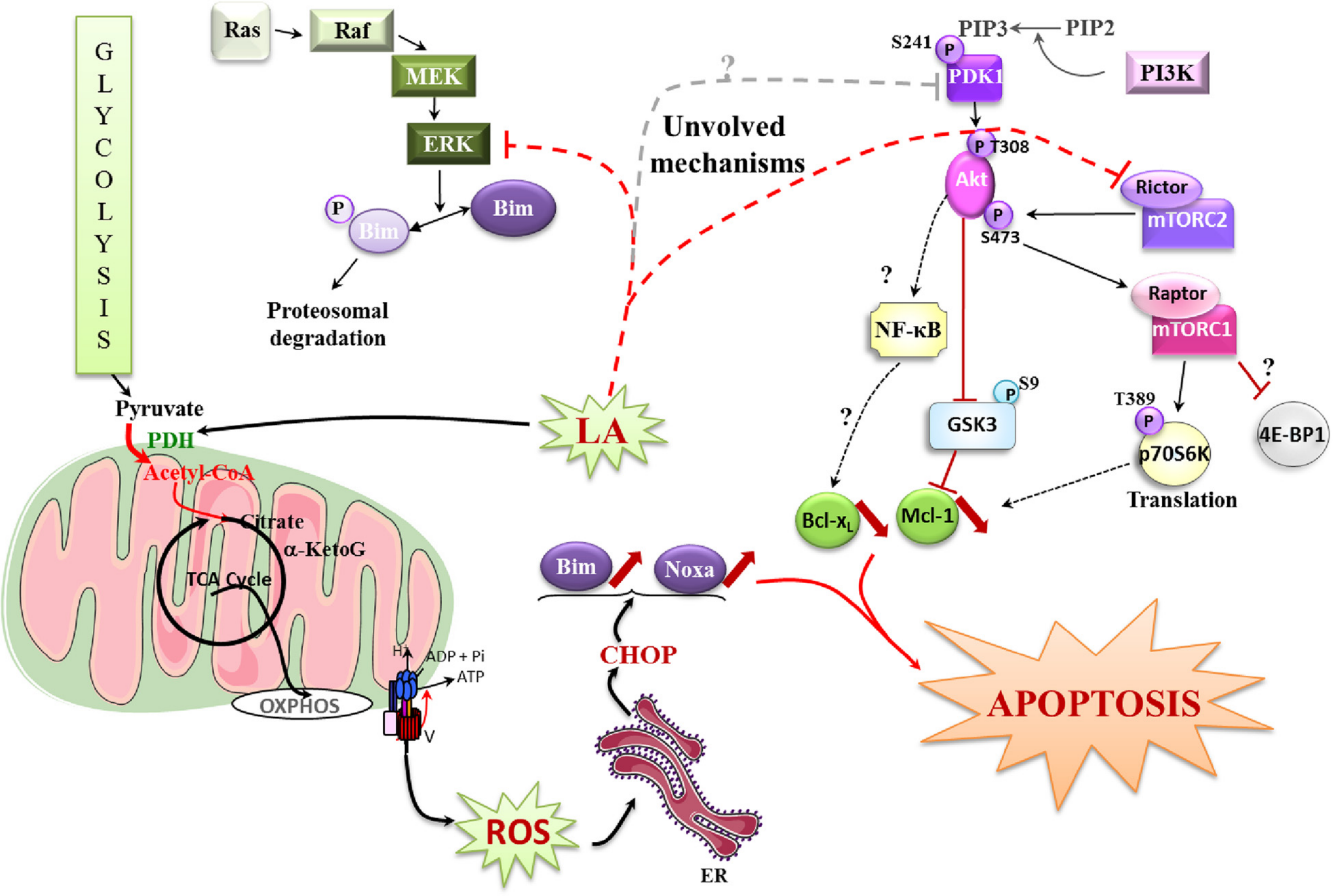
Schematic representation of the role of lipoic acid in sensitive or chemoresistant ovarian carcinoma cells. LA induces major apoptosis by up-regulation of Bim associated with down-regulation of Bcl-x_L_ and Mcl-1. In explaining the Bim induction, the role of CHOP is highly suggested. CHOP induction would result from the over production of ROS induced by the reconnection of the TCA cycle and glycolysis induced by LA. In the metabolism of cancer cells, a disconnection between glycolysis and TCA cycle favours cancer cells growth. Lipoic acid reactivates PDH activity and thus, reconnects the glycolysis to the TCA cycle. This process leads to cell death presumably by generating oxidative stress characterized by ROS production. ROS promote ER stress; a process inducing CHOP expression. This expression upregulates the BH3 only pro-apoptotic factor Bim. On the other hand, LA is involved in the inhibition of Bcl-x_L_ and Mcl-1, two major anti-apoptotic proteins, which conspire with bim to induce major apoptotic death. We suppose that the inhibition of Bcl-x_L_ and Mcl-1 translates by the inactivation of PI3K/Akt/mTOR and ERK pathways, characterized by a decrease of various targets (such as PDK1, AKT, mTORC1, p70S6K…) leading to activation of GSK3. These assumptions which remain to be studied, are shown in blurred. Abbreviations: Acetyl-CoA: acetyl coenzyme a, α-KetoG: α -Ketoglutarate, OXPHOS: oxidative phosphorylation, ER: endoplasmic reticulum, Bim: Bcl-2 interacting mediator of cell death, PI3K: Phosphoinositide 3 kinase, PIP2: Phosphoinositide (3,4) biphosphate, PIP3: Phosphoinositide (3,4,5) triphosphate, mTORC1 or 2: mammalian target of rapamycin complex 1 or 2, Akt: protein kinase B, p70S6K: p70 S6 kinase, 4-EBP1: 4-E binding protein 1, GSK3: glycogen synthase kinase 3, NF-κB: nuclear factor-kappaB, Mcl-1: myeloid cell leukemia sequence 1, Ras: Rous Avian Sarcoma, MEK: MAPK/ERK kinase, ERK: extracellular signal-regulated MAP kinase

In summary, we demonstrated that LA induced massive apoptotic cell death through inhibition of the two anti-apoptotic proteins Mcl-1 and Bcl-x_L_, and induction of the pro-apoptotic BH3-only protein Bim in two human ovarian cancer cells (one sensitive and one resistant to cisplatin). We found the induction of Bim was crucial in this process, because its blockage by siBim drastically reduced apoptosis. We showed that LA promoted massive cell death through an increase of ROS, a phenomenon that could lead to Bim, Noxa through CHOP induction (Fig. 6). The pan-inhibitor effects of LA could be further investigated and incite to test this molecule *in vivo* studies [11, 54].

## Abbreviations

CHOP: C/EBP homologous protein (CHOP)
LA: Lipoic acid
PDK1: Phosphoinositide-dependent kinase-1
PERK: protein kinase RNA-like endoplasmic reticulum kinase
PDH: Pyruvate dehydrogenase
PFK1: Phosphofructokinase 1
NAC: N-acetylcysteine
ROS: Reactive oxygen species
SDH: Succinate dehydrogenase
TCA: Tricarboxylic acid cycle
